# ROS-Mediated Therapeutic Strategy in Chemo-/Radiotherapy of Head and Neck Cancer

**DOI:** 10.1155/2020/5047987

**Published:** 2020-07-22

**Authors:** Gan Huang, Shu-Ting Pan

**Affiliations:** Department of Oral and Maxillofacial Surgery, The First Affiliated Hospital of Nanchang University, Nanchang, 330006 Jiangxi, China

## Abstract

Head and neck cancer is a highly genetic and metabolic heterogeneous collection of malignancies of the lip, oral cavity, salivary glands, pharynx, esophagus, paranasal sinuses, and larynx with five-year survival rates ranging from 12% to 93%. Patients with head and neck cancer typically present with advanced stage III, IVa, or IVb disease and are treated with comprehensive modality including chemotherapy, radiotherapy, and surgery. Despite advancements in treatment modality and technique, noisome recurrence, invasiveness, and resistance as well as posttreatment complications severely influence survival rate and quality of life. Thus, new therapeutic strategies are urgently needed that offer enhanced efficacy with less toxicity. ROS in cancer cells plays a vital role in regulating cell death, DNA repair, stemness maintenance, metabolic reprogramming, and tumor microenvironment, all of which have been implicated in resistance to chemo-/radiotherapy of head and neck cancer. Adjusting ROS generation and elimination to reverse the resistance of cancer cells without impairing normal cells show great hope in improving the therapeutic efficacy of chemo-/radiotherapy of head and neck cancer. In the current review, we discuss the pivotal and targetable redox-regulating system including superoxide dismutases (SODs), tripeptide glutathione (GSH), thioredoxin (Trxs), peroxiredoxins (PRXs), nuclear factor erythroid 2-related factor 2/Kelch-like ECH-associated protein 1 (Nrf2/keap1), and mitochondria electron transporter chain (ETC) complexes and their roles in regulating ROS levels and their clinical significance implicated in chemo-/radiotherapy of head and neck cancer. We also summarize several old drugs (referred to as the non-anti-cancer drugs used in other diseases for a long time) and small molecular compounds as well as natural herbs which effectively modulate cellular ROS of head and neck cancer to synergize the efficacy of conventional chemo-/radiotherapy. Emerging interdisciplinary techniques including photodynamic, nanoparticle system, and Bio-Electro-Magnetic-Energy-Regulation (BEMER) therapy are promising measures to broaden the potency of ROS modulation for the benefit of chemo-/radiotherapy in head and neck cancer.

## 1. Introduction

Head and neck cancer (HNC) is the seventh most frequently occurring malignancy worldwide in 2018 (accounting for 4.9% of all cancer sites) [1]. It is reported that lip, oral cavity, and pharyngeal cancers could be responsible for the 529,500 new cancer cases (accounting for about 3.8% of all cancer cases) and the 292,300 cancer-related deaths (accounting for about 3.6% of all cancer deaths) in 2012 globally, and the incidence is predicted to increase by 62% to 856,000 cases in 2035 [2]. Due to the tenacious resistance of cancer cells to therapy, the five-year survival rate has not been significantly improved during past decade [3]. Commonly used radiation and chemotherapy drugs affect the prognosis of HNC through reactive oxygen species (ROS) regulation directly and indirectly [4]. The balance of cellular ROS is levered by ROS generators including mitochondrial ROS, NADPH oxidases, and other enzymes and ROS eliminators such as superoxide dismutases (SODs), tripeptide glutathione (GSH), and nuclear factor erythroid 2-related factor 2/Kelch-like ECH-associated protein 1 (Nrf2/Keap1) [5]. ROS has been implicated in cancer initiation, formation, and development as well as therapy resistance [6]. In spite of some inspiring clinical trials concerning ROS modulation in comprehensive treatment of HNC, the personalized treatments call for multiple therapeutic strategies. During the past years, genetic or pharmaceutic methods for modulating ROS in HNC are showing great preclinical and clinical significance in the combined modality of chemo-/radiotherapy. Ongoing researches from other groups and our own are making efforts in modulating the cellular ROS level to enhance the efficacy of chemo-/radiotherapy and to decrease side effects and toxicity without compromising therapeutic efficacy in the treatment of HNC.

## 2. The Epidemiology of Head and Neck Cancer and Leading Therapeutic Challenges

**Figure 1:**
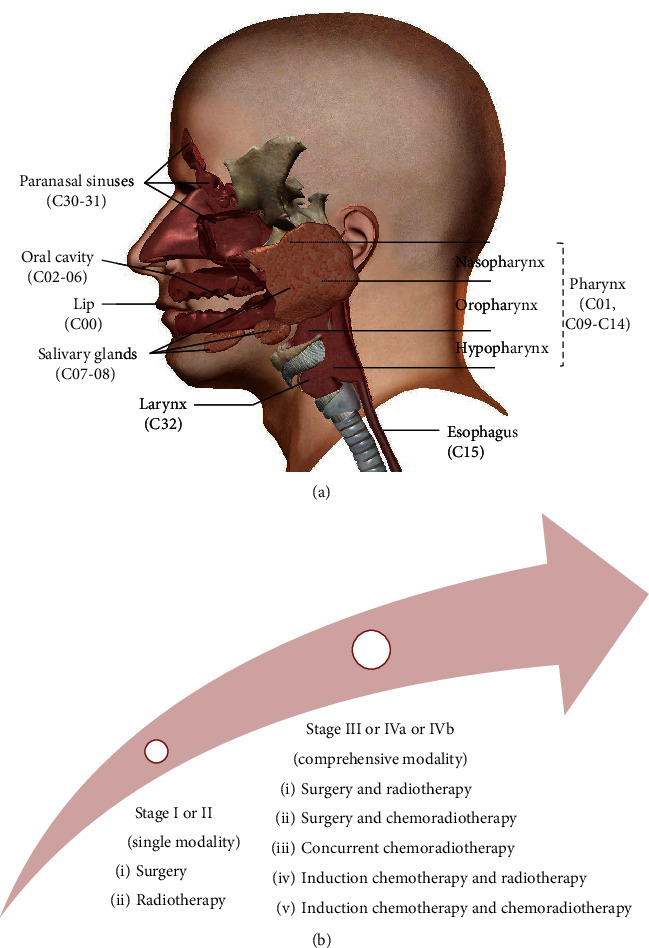
Anatomical sites and treatment of HNC. (a) Head and neck cancers incorporate multiple anatomical regions concerning the lip (C00), oral cavity (C02-06), salivary glands (C07-08), oropharynx (C01, C09-C10), nasopharynx (C11), hypopharynx (C12-14), esophagus (C15), paranasal sinuses (C30-31), and larynx (C32). International Classification of Diseases 10th revision, website: http://www.who.int/classifications/icd/icdonlineversions/en/. (b) HNC patients with early stages (stages I and II) are recommended for single modality including surgery or radiotherapy. Comprehensive modality including surgery, radiotherapy, and chemotherapy is guided for advanced cases (stages III, IVa, and IVb). *Note*. NCCN Clinical Practice Guidelines in Oncology: Head and Neck Cancers, website: https://www.nccn.org.

Head and neck cancer incorporates multiple organs from complex anatomical topographies which include the lip (C00), oral cavity (C02-06), salivary glands (C07-08), oropharynx (C01, C09-C10), nasopharynx (C11), hypopharynx (C12-14), esophagus (C15), paranasal sinuses (C30-31), and larynx (C32) [1, 2, 7–9] (Figure 1(a)). About 85-90% of HNC are squamous carcinoma that originated from epithelial cells (HNSCC) [9, 10]. There are more than 800,000 new cases and 500,000 deaths of esophageal, lip, oral cavity, and nasopharyngeal cancers worldwide [11, 12]. In 2020, there are 84,070 estimated new cases and 30,670 estimated deaths of HNC in the United States. The oral cavity and pharynx cancers rank first among the new cases of HNC, while they rank eighth (4%) among all cancer sites in males. The esophageal cancers top the list of HNC mortality [13]. In general, males are more inclined to suffer from HNC [1]. Advancing age is a disadvantage to HNC prognosis. HPV status of HNC influences the therapeutic outcome; HPV-positive HNC are associated with a better response to chemotherapy and radiotherapy even with stage IV disease [8, 14]. The five-year survival rates of HNC range from 12% to 93% from among different ages, gender, educational levels, race, and geographical locations as well as different cancer sites, pathological grades, and received therapy [2, 3, 12, 15, 16].

Due to the special anatomical position, HNC are prone to exert a negative impact on language, respiration, eating, swallowing, and digestion. Besides, rapid blood supply and lymphatic regurgitation render HNC to be inclined to cervical lymph node metastasis [14]. The treatment strategy depends on individual factors concerned with the site of the cancer and tumor/node/metastasis (TNM) stage, as well as patient preference [14, 16]. In general, HNC at an early stage (TNM: I and II) are well controlled after surgery or radiotherapy. HNC at an advanced stage (TNM: III, IVa, and IVb) are locally invasive and accompanied by metastasis of cervical lymph nodes. It is difficult to completely remove the cancer. They call for comprehensive treatment of surgery, radiotherapy, and chemotherapy to reduce the original lesion or control the postoperative period [17–19] (Figure 1(b)). Unfortunately, two-thirds of patients with HNC are advanced cases (T3-T4 and/or cervical adenopathy) when they are first examined, at which point they have lost the optimum time for surgery [20]. Cisplatin- (CDDP-) based chemotherapy and adjuvant radiotherapy are still the first-line treatment options for advanced patients [14]. In spite of the advancement of diagnosis and treatment modality, such as minimally invasive transoral surgery, intensity-modulated radiotherapy (IMRT), gene-targeting drugs (anti-EGFR therapy), and immunotherapy (anti-PD-1 therapy) of HNC, the long-term survival rate of patients is not substantially improved [21]. Disappointingly, more than 50% of patients develop recurrence in either local or distant sites within two years of treatment [22, 23]. Recurrence and posttherapy complications (marrow depression, immune suppression, muscle fibrosis, renal toxicity, mucosal damage, salivary gland secretion disorders, mandibular fractures, and necrosis) severely affect the quality of life and lead to a high morbidity of HNC patients [8, 24]. Resistance to treatment is correlated with recurrence and morbidity. Thus, developing new treatment strategies to surmount recurrence and complications is vital for improving the long-term survival and quality of life of patients with HNC [25, 26]. Cancer cells are prone to increase oxidative stress and switch the metabolism pattern to aerobic glycolysis called the Warburg effect [27–29]. Targeting these unique biochemical alterations in cancer cells might be a feasible strategy to prevent therapy resistance and ameliorate the prognosis [30].

## 3. Redox Adaptation in Cancer Cells and Its Implicated Modulation in Chemo-/Radiotherapy of HNC

**Figure 2:**
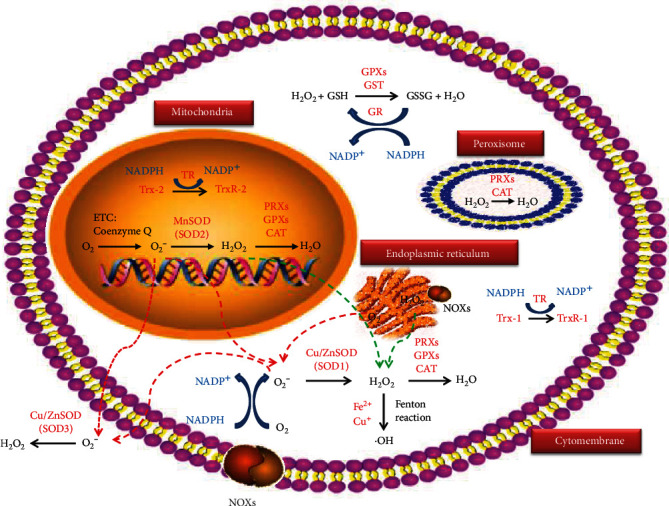
ROS sources and antioxidant systems. Mitochondrial respiration ETC and the membrane-bond NOX complexes are the two major ROS resources. Leakage of electrons from ETC is mediated by coenzyme Q and produces O_2_^−^ through O_2_. There are three isoforms of SODs to defend oxidation. Cu/Zn SOD (SOD1) in the cytoplasm, MnSOD (SOD2) in the mitochondria, and Cu/Zn SOD (SOD3) in the extracellular matrix can rapidly convert O_2_^−^ to H_2_O_2_. NOXs catalyze the generation of O_2_^−^ from O_2_ and NADPH. H_2_O_2_ is converted to toxic ·OH by a metal (Fe^2+^ or Cu^+^) catalyst through the Fenton reaction. H_2_O_2_ can be converted into H_2_O by PRXs, GPXs, and CAT. Besides, Trxs (the cytoplasmic Trx-1 and the mitochondrial Trx-2) can reduce oxidized PRXs. Trxs themselves are also reduced to TrxR by TR using NADPH as an electron donor. GPXs oxidize reduced GSH to GSSH. GSSH is reduced back to GSH by GR accompanied by an electron from NADPH. *Note*. ETC: electron transport chain; NOXs: NADPH oxidase; SODs: superoxide dismutases; H_2_O_2_: hydrogen peroxide; NADPH: nicotinamide adenine dinucleotide phosphate; ·OH: hydroxyl radicals; PRXs: peroxiredoxins; GPXs: glutathione peroxidases; CAT: catalase; Trx: thioredoxin (oxidized); Trx-R: thioredoxin (reduced); TR: thioredoxin reductase; GSH: tripeptide glutathione (reduced); GSSH: glutathione disulfide (oxidized); GR: glutathione. Green dotted lines denote H_2_O_2_ diffusion. Red dotted lines denote O_2_^−^ diffusion.

Reactive oxygen species (ROS) is a term that denotes a series of intermediate products produced during the oxidative metabolism of cells, including two-electron (nonradical) ROS such as hydrogen peroxide (H_2_O_2_), organic hydroperoxides (ROOH), singlet molecular oxygen (^1^O_2_), hypochlorous acid (HOCl), hypobromous acid (HOBr), and ozone (O_3_); free radical ROS include the superoxide anion radical (O_2_^−^), the hydroxyl radical (·OH), the peroxyl radical (ROO·), and the alkoxyl radical (RO·) [5]. Mitochondrial electron transport chain (ETC) complex [31] and nicotinamide adenine dinucleotide phosphate oxidases (NOXs) [32] are the major endogenous sources of ROS. To protect lipids, proteins, and nucleic acids from indiscriminate damage induced by free radicals, cells arrange a complex network of antioxidant systems to maintain genomic stability and proper cellular function [6]. SODs and GSH are the predominant antioxidant systems [30] (Figure 2). Other ROS generators including cytochrome p450, lipoxygenase, and xanthine oxidase and scavengers such as catalase (CAT), peroxiredoxins (PRXs), glutathione peroxidases (GPXs), vitamin C, and vitamin E closely participate in the redox system [6, 33]. Nrf2/Keap1 complex regulates redox hemostasis by sensing oxidative stress and then activating downstream antioxidant elements such as glutathione-S-transferases (GST), NAD(P)H:quinone oxidoreductase (NQO1), PRXs, GPXs, and CAT [34–36]. Other redox-sensitive transcription factors such as nuclear factor-*κ*B (NF-*κ*B), p53, and hypoxia inducible factor 1 (HIF-1) lead to elevation of ROS-eliminating enzymes like SOD and GSH, activating survival factors such as myeloid cell leukaemia-1 (Mcl-1) and B-cell lymphoma-2 (Bcl-2), and inhibition of cell death factors [30].

**Figure 3:**
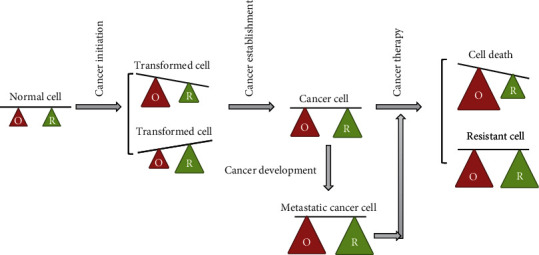
Redox adaptation in cancer formation, development, and therapy. Cellular redox homeostasis is maintained by ROS generation and elimination balance in normal cells. Once continuous exogenous stimulus and endogenous oncogene activation disrupt the balance, either a high level of ROS is produced or antioxidants are excessively enhanced, and cancer cells are hence formed. In order to survive oxidative stress, these cancer cells regain redox homeostasis via multiple mechanisms such as increasing ROS-scavenging enzymes. During the development of cancer and even during the process of therapy resistance, the cancer cells gradually enhance both ROS level and antioxidant enzymes. Thus, abrogating the adaptation mechanisms by increasing the ROS level beyond a threshold that is incompatible for cellular survival and attenuating antioxidant defense systems can be an attractive strategy to kill cancer cells and thus reverse resistance and limit cancer progression. *Note*. O: oxidative status; R: reducing status.

In normal cells, redox balance is well orchestrated via antioxidant defense systems. Once exposed to continuous exogenous stimuli such as radiation and carcinogens and endogenous oncogene activation such as *H-Ras*, the normal cells fail to leverage the redox balance, thus forming cancer cells [37]. To adapt to the oxidative stress, the initiated cancer cells will tactfully enhance the antioxidant enzymes accordingly. Consequently, both the ROS level and ROS-scavenging enzymes are increased to benefit cancer cell survival, metastasis, and even resistance [6, 38]. In other words, ROS represents a double-edged sword [39]. Basal levels of ROS can maintain the homeostasis of normal cells; chronic and low levels of ROS promote cell mitosis and increase genomic instability to induce the occurrence and progression of tumors; moderate concentrations of ROS cause temporary or permanent cell cycle arrest and may induce cell differentiation [39]; acute and high concentrations of ROS damage macromolecules and thus induce apoptosis, necrosis, and ferroptosis [40]. Therefore, the high concentration of ROS in cancer cells and the defects of their antioxidant damage defense system render cancer cells more susceptible to ROS modulation. In the case of the same concentration of ROS, cancer cells first undergo apoptosis while normal cells can tolerate it [41–43]. Adjusting intracellular ROS levels to efficiently kill cancer cells and reduce the side effect of chemo-/radiotherapy is currently considered as the fundamental means of cancer treatment [30, 40, 44] (Figure 3).

**Figure 4:**
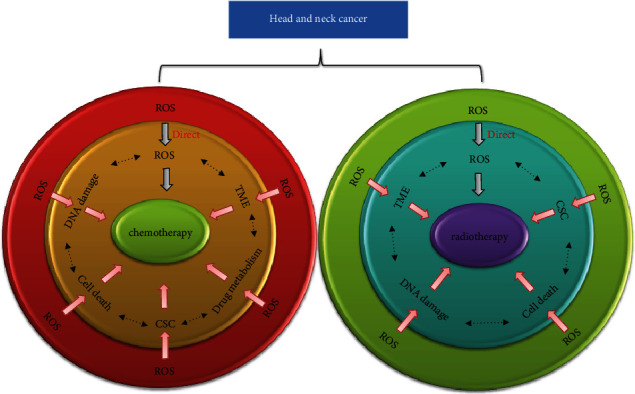
ROS is implicated in the modulation in the chemo-/radiotherapy of HNC. ROS can directly and indirectly affect the efficiency of chemotherapy drugs such as cisplatin and 5-Fu and/or radiation therapy in HNC. A direct effect is seen in terms of ROS-induced lethal genetic damage. Indirect mechanisms include cell death regulation such as apoptosis and autophagy, DNA damage repair, drug metabolism, cancer stem cell (CSC) characteristics, and tumor microenvironment (TME) which are modulated by ROS in the chemotherapy of HNC. Radiotherapy exerts its function through induction of DNA damage within the cell. Except for drug metabolism, other mechanisms are all involved in ROS-mediated radiotherapy efficacy in HNC. Because of the dual role of ROS, the complex modulation network can adapt towards the killing effect of cancer cells or readapting the therapy stimuli. Generally, low and chronic ROS may call for more antioxidant stress defense to protect cancer cells, while high and acute ROS may kill cancer cells with no margin for adaptation. *Note*. ROS: reactive oxygen species; HNC: head and neck cancer; 5-Fu: 5-flurouracil; DNA: deoxyribonucleic acid; CSC: cancer stem cell; TME: tumor microenvironment.

During chemotherapy and/or radiotherapy in HNC, frequent resistance and accompanying side effects are the head-scratching puzzles. Despite the development of gene-targeted drugs such as bortezomib, sorafenib, and cetuximab for the treatment of HNC, evasion of therapy remains the main obstacle to cure [21]. The implicated regulation of ROS is of great significance for cancer treatment, because commonly used radiation and chemotherapy drugs affect the prognosis of HNC through ROS regulation directly and indirectly [4] (Figure 4). Physiological mechanisms which mediate the chemotherapy efficacy by ROS are as follows: (1) cell death regulation [45–48], (2) deoxyribonucleic acid (DNA) damage repair [49–51], (3) drug metabolism [30, 52, 53], (4) tumor microenvironment [54, 55], and (5) cancer stem cell (CSC) characteristics [56]. In radiation biology, an “oxygen effect” is an important phenomenon which refers to the enhanced killing effect in the presence of oxic conditions. Irradiation exposure can induce mitochondrial-dependent ROS generation [57]. ROS-modulated DNA damage repair [58–61], cell death regulation [62–65], tumor microenvironment [66, 67], and CSC characteristics [67, 68] greatly affect the radiotherapy efficiency. Among these biological factors, cell death and DNA damage are the most common aspects regulated by cellular redox status.

Currently, it is recognized that CSC presenting self-renewal and pluripotent differentiation capabilities are more inclined to obtain heterogeneous, aggressive, and resistant phenotypes [69, 70]. Especially in poorly vascularized hypoxic tumor niches, CSC characteristics can be well maintained with a high level of ROS-eliminating enzymes, drug resistance transporter proteins, DNA repair enzymes, and antiapoptotic proteins such as Bcl-2 [71, 72]. A lower ROS concentration is found in CSC-enriched populations from irradiated head and neck cancers, compared with nontumorigenic cells [73]. With prolonged exposure to low oxygen levels, CSC cells may undergo epithelial-to-mesenchymal transition (EMT) and acquire the ability to invade and metastasise to local lymph nodes and distant organs [71]. ROS have been implicated in EMT via the activation of EMT-inducing transcription factors including Snail/Slug, ZEB1/2, Twist1/2, HIF-1, and Dlx-2 by modulating upstream signaling pathways such as epidermal growth factor (EGF), Wnt/*β*-catenin, transforming growth factor-*β* (TGF-*β*), mitogen-activated protein kinase (MAPK), phosphatidylinositol 3-kinse/protein kinase B (PI3K/Akt), Hedgehog, and Notch [68, 74, 75]. Moreover, EMT is closely linked to CSC and the metabolic alteration of cancer cells to avoid hostile environments [76, 77]. Tumor cell-derived low level of ROS inhibits caveolin-1 expression in cancer-associated fibroblasts (CAFs) which is implicated in the stabilization and nuclear accumulation of EMT-inducing transcription factor HIF-1 [78, 79]. The tightly oxygen-regulated subunit HIF-1*α* effectively induces angiogenic genes such as VEGF [80] and shifts glucose metabolism from aerobic respiration to anaerobic glycolysis via transactivation of glucose transporter GLUT-1 and lactate dehydrogenase (LDH) [81, 82]. An enhanced HIF-1*α* level has been observed in the CSC subpopulation of HNSCC [67] and linked to poor prognosis and resistance to chemotherapy and radiotherapy [83, 84]. Pharmacological depletion of ROS scavengers reduces the colony-forming capacity of CSC and then increases the radiosensitivity of HNC [73]. Moreover, the capacity of cellular ROS to sensitize the chemo-/radiotherapy of cancer cells depends on the basal level of ROS in such cells. Below a certain threshold, ROS can facilitate survival, but if a certain limit is broken through, cells will die due to intolerance [40]. Adjusting the appropriate ROS level can synergize conventional therapy while reducing the dosage of chemotherapeutic drugs and/or radiation in the clinical condition and thereby alleviating the potential side effects.

In view of the strong reactivity, short life, and opposing roles of ROS, specific quantification and localization of ROS are an important cornerstone for a thorough understanding of its role in cancer initiation, development, and therapy. There are small molecule probes and gene-encoded probes designed to detect whole-cell ROS and mitochondrial ROS. The advantages and disadvantages of these probes are listed in Table 1. Only by clearly understanding the characteristics and defects of these probes can we obtain the accurate research outcomes concerning cellular stress response and therapeutic dose. Besides, methods designed for real-time monitoring of the kinetic changes in the cellular redox state in vivo may further facilitate a comprehensive understanding of the mechanisms of redox biology [85].

## 4. Modulate ROS Generation and Elimination to Improve the Efficacy of Chemo-/Radiotherapy in HNC

Once cancer cells are exposed to chemotherapy, radiation, and other treatments, the readaption of the redox status is launched. This in turn provides us a platform to modulate ROS scavengers and generators in order to improve the efficacy of chemo-/radiotherapy.

### 4.1. Targeting the SOD Antioxidant System in HNC

Superoxide dismutases (SODs) are the main antioxidants which can rapidly and specifically convert O_2_^−^ to hydrogen peroxide (H_2_O_2_). Three isoforms of SODs are found in mammals: SOD1 (Cu/ZnSOD) in the cytoplasm, SOD2 (MnSOD) in the mitochondria, and SOD3 (Cu/ZnSOD) in the extracellular matrix [6]. Noteworthy, the homotetramer SOD2 (MnSOD) which is the most researched SOD in cancer is found exclusively in the mitochondrial matrix [99]. MnSOD acts as a double-edged sword in cancer development [100]. Some researches show that the expression level of MnSOD is decreased compared with normal tissues in breast cancer, pancreatic cancer, and ovarian cancer [101–103]. On the contrary, other researches reveal the higher expression of MnSOD in the malignant progression of gastric cancer, lung cancer, and esophageal cancer [104–106]. During radiation, MnSOD plays a vital role modulating cellular redox balance towards the good and bad sides known as radiosensitization and redioresistance [107]. This dual effect may be ascribed to differences in the expression and/or activity of other antioxidant enzymes like GSH/GSSH, thioredoxins, and catalases in different types of cancers.

SOD mimics such as MnTnBuOE-2-PyP^5+^ (BMX-001) and Mn (II) pentaaza macrocycle (GC4419) possessing high SOD-like activity show great hope in multiple clinical applications [108]. Ashcraf et al. found that MnTnBuOE can alleviate mucositis (manifested as xerostomia and fibrosis in salivary glands) induced by radiation in non-tumor-bearing female C57BL/6 mice with a dose-modifying factor of 0.77. Human pharyngeal squamous carcinoma cell FaDu xenograft nude mice treated with a combination of RT and MnBuOE showed greater radiosensitivity than a single RT group. The dose adjustment factor is analyzed as 1.3 [109]. Another report from this team discovered that lower doses of MnBuOE mitigated cisplatin-induced oral ulcer formation, bleeding, and furfuration in the radiation area. MnBuOE did not meddle with RT/cisplatin-regulated neoplasm growth [110]. BMX-001 is undergoing phase I clinical trials concerning its safety and pharmacokinetic and radiation protection in conditions of locally advanced head and neck cancer (clinical trial number: NCT02990468).

A randomized, double-blind phase IIb clinical trial of the effects of GC4419 on radiation-induced mucositis in patients with head and neck cancer was completed on 29 August 2019. 223 patients with HNC from 44 institutions who were planning to receive definitive or postoperative IMRT plus cisplatin were randomly allocated into the 30 mg GC4419, 90 mg GC4419, and placebo groups. The outcomes are inspiring. Compared with the placebo group, 90 mg GC4419 treatment showed a decreasing incidence, duration, and severity of oral mucositis induced by 60-72 Gy IMRT (at least two oral mucosal sites) and concurrent cisplatin. No significant toxicity specified or enhanced by GC4419 in IMRT plus cisplatin treatment was observed. A phase III clinical trial (clinical trial number: NCT03689712) to investigate the effects of GC4419 on radiation-induced oral mucositis in patients with head and neck cancer is currently in progress [111].

### 4.2. Targeting the GSH Antioxidant System in HNC

Tripeptide Glutathione (GSH) is an important intracellular antioxidant that powerfully transfers hydrogen peroxide to H_2_O and plays a role in the detoxification of many peroxides and electrophilic compounds [112]. Cysteine-glutamate antiporter (System xc^−^; xCT) encoded by SLC7A11 acts as cysteine importer to the cellular ROS which is essential for GSH biosynthesis [113]. Glutamate-cysteine ligase (GCL) synthesizes substrate cysteine, glycine, and glutamate to GSH [6]. That is to say, cysteine availability and GCL activity determine the synthesis of GSH. GPXs and GST oxidize reduced GSH to glutathione disulfide (GSSG). GSSG can be reduced by glutathione reductase (GR) back to GSH [114]. Meanwhile, nicotinamide adenine dinucleotide phosphate (NADPH) serves as an electron donor [115]. The ratio of reduced and oxidized glutathione (GSH : GSSG) is a representative indicator of cell antioxidant capacity. The imbalance in the synthesis and conversion of GSH is widely implicated in Parkinson's disease [116], cystic fibrosis [117], skin whitening [118], diabetes [119], and schizophrenia [120] as well as cancer [112, 121].

Increased GSH has long been considered as an accomplice in the progression and multidrug resistance of cancer [122–126]. GSH depletion obtained by the irreversible GCL inhibitor BSO is the most commonly used method and is associated with many chemotherapy drugs. However, previous phase I clinical trials concerning the anticancer effect of GSH inhibitor buthionine sulfoximine (BSO) were unsatisfactory [127, 128]. Shortcomings such as a short half-life and nonselective GSH depletion on normal cells limited its clinical application. Over the past two decades, BSO stood at a standstill and did not proceed to Phase II clinical trials. Based on this, researchers carried out a large amount of work with respect to GSH analogues [129] or a combination treatment with other antioxidant systems [130]. Key elements such as GST and xCT in the GSH synthesis process are also excavated to solve chemoresistance [125]. Telcyta (TLK-286), a GSH analogue, has completed the phase II/III clinical trials concerning its treatment efficacy combined with cisplatin, carboplatin, doxorubicin, paclitaxel, and docetaxel in several types of locally advanced or metastatic or refractory resistant cancers (https://www.clinicaltrials.gov/). However, HNC are not covered in these trials. The clinical application of TLK-286 in HNC is hence not suggested in the latest NCCN and ASCO guidelines [17, 19].

There are some preclinical researches in the matter of the GSH antioxidant system in HNC. The combination of BSO and the thioredoxin reductase (TrxR) inhibitor auranofin can synergistically sensitize erlotinib-induced cell death of HNC in vitro and in vivo [131]. On the other hand, the BSO and auranofin combination can simultaneously activate the Nrf2-antioxidant response element pathway which may lead to suboptimal GSH and Trx inhibition in resistant HNC. Thus, inhibition of Nrf2 is proven to make the anticancer effect of BSO and auranofin back to the optimum for HNC [130].

Ethacrynic acid (ECA), a GST inhibitor, was designed to be a methoxy poly(ethylene glycol)-poly(lactide)-disulfide bond-methacrynic acid (MPEG-PLA-SS-ECA) nanoparticle drug carrier, which encapsulates pingyangmycin (PYM) or carboplatin (CBP) separately. The PYM- and CBP-resistant oral squamous cell carcinoma cell lines SCC15/PYM and SCC15/CBP were established to examine the reversal effect of drug resistance by the MPEG-PLA-SS-ECA/PYM and MPEG-PLA-SS-ECA/CBP nanoparticle. The resistant factor values of MPEG-PLA-SS-ECA/PYM and MPEG-PLA-SS-ECA/CBP nanoparticles in SCC15/CBP and SCC15/PYM cells were 1.51 and 1.24. Effective inhibition of GST by nanoparticles shows great hope in reversing PYM and CBP drug resistance in oral cancer [132]. These findings are expected to proceed to further clinical trials.

### 4.3. Targeting the Trx Antioxidant System in HNC

The thioredoxin (Trx) system is a disulfide reductase system widely existing in many species from prokaryotes to mammals. It is composed of Trx, thioredoxin reductase (TrxR), coenzyme *α*-NADPH, and Trx-interacting protein (TXNIP) [133]. The predominant location is the cytoplasm containing Trx-1 and TrxR-1, and the subordinate location is mitochondria containing Trx2 and TrxR-2 [134]. Trx with a conserved redox catalytic site (-Cys-Gly-Pro-Cys-) can affect multiple biological functions such as intracellular redox regulation, DNA synthesis, selenium metabolism, cell growth regulation, and apoptosis [135]. TrxR is the only known enzyme capable of reducing Trx, which regulates the protein's thiol/disulfide bond balance by disulfide reductase activity. The dynamic balance between TrxR reduction ability and oxidative stress is the key factor to ensure body homeostasis [130, 136]. Elevated levels of Trx system proteins (Trx-1, TrxR-1, Trx-2, and TrxR-2) and decreased levels of TNXIP protein are involved in various cancers [137–140]. A similar phenomenon was discovered in oral cancers [141–143] and esophageal adenocarcinoma [144]. Moreover, Kaplan-Meier's analysis revealed that the expression level of Trx was significantly related with a lower 5-year survival rate in patients with tongue squamous cell carcinoma [141]. The expression level of TrxR-1 in HPV^−^ cells is much higher than in HPV^+^ cells in HNSCC. This leads to intrinsic resistance to radiation in HPV^−^ cells [145]. Trx inhibitors such as 1-methylpropyl 2-imidazolyl disulfide (PX-12), 4-benzothiazole-substituted quinol (PMX464), and suberoylanilide hydroxamic acid (SAHA) exert anticancer activity by ROS generation, cell cycle arrest, and apoptosis induction via MAPK signaling pathways [136]. SAHA can synergize the killing effect of bortezomib in EBV-positive nasopharyngeal carcinoma (NPC) HK1-EBV, HONE1-EBV, HA, and C666-1 cell lines. In vivo, bortezomib and SAHA effectively induced apoptosis and inhibited the growth of NPC xenografts in nude mice. ROS generation and subsequent induction of apoptosis indicated by elevated levels of cleaved caspases 3, 7, and 9 and cleaved PARP are the key mechanisms for this synergistic effect [146].

### 4.4. Targeting the PRX Antioxidant System in HNC

Peroxiredoxins (PRXs) are a family of 22-27 kDa non-selenium-dependent glutathione peroxidases that catalyze the reduction of H_2_O_2_ and peroxynitrite (ONOO^−^). There are six subtypes of Prxs (Prx I-VI) found in mammals [147]. PRXs participate in the occurrence and development of tumors by regulating the level of redox inside and outside the mitochondria [148]. Prx1 was observed to be significantly increased in ESCC clinical tissue samples [149]. Activation of the mTOR/p70S6K pathway is involved in Prx1-promoted tumorigenesis [150]. Another study discovered that Prx II was greatly augmented in patients who failed to respond to chemotherapy or radiation therapy. And in head and neck cancer UMSCC-11A cells, the expression level of Prx II was elevated after 3 Gy radiation or treatment of cisplatin (5 mg/ml) and 5-flurouracil (5-Fu) (2.5 mg/ml). The antisense of PrxII could be sensitized to radiation or chemotherapy inducing apoptosis in UMSCC-11A cells [151]. In a recent study, the expression level of Prx6 was analyzed by immunohistochemistry in 95 ESCC samples and 26 paired adjacent normal tissues. Prx6 was upregulated in ESCC tissues and correlated with the elevated proliferation markers such as Ki67, PCNA, and CyclinD1. Silencing Prx6 greatly inhibited the proliferation of Eca-109 and TE-1, while the overexpression of Prx6 facilitated the migration and invasion of Eca-109 and TE-1 via elevating the Akt and Erk1/2 signaling pathway. Moreover, the downregulation of Prx6 synergizes the apoptosis induced by 8 Gy X-ray irradiation. These findings are further validated in the ESCC xenograft mode in vivo. Inhibition of Prx6 shows a novel therapeutic strategy for radiosensitization in ESCC [152].

### 4.5. Targeting the Nrf2/Keap1 Antioxidant System in HNC

Nrf2 and Keap1 are the major proteins that coordinate the induction and transcription of various antioxidant enzymes [34]. Under normal physiological conditions, Nrf2 binds to the Keap1/CUL3/RBX1 E3-ubiquitin ligase complex in large amounts and degrades rapidly in the cytoplasm. When the oxide accumulates, Nrf2 and Keap1 dissociate and transfer or bind to antioxidant enzymes in the promoter region of detoxification phase II enzymes, such as NQO1, GST, glutathione peroxidase (Gpx), peroxidase I, glutathione ligase, glutathione, epoxide hydrolase, and heme oxygenase (HO-1). These enzymes can protect the body from active substances (such as ROS) and some toxic substances [34, 35]. A large number of studies have shown that Nrf2 is related to the occurrence of metabolic disorders and cancer initiation, and these are well reviewed by Cuadrado et al. and Rojo de la Vega et al. [153, 154]

Nrf2 gene (NFE2LE) mutations are a mechanism of Nrf2 activation which has been correlated with poor survival [155]. Besides, a high frequency (60%) of DNA level inactivation to the Nrf2 inhibitor Keap1/CUL3/RBX1 E3-ubiquitin ligase complex is related to HNSCC. And this complex disruption is unique to HNSCC. The median survival rate was decreased when the altered complex increased. Nrf2 activation is an underlying prognostic indicator in HNSCC [156].

A recent retrospective study concerning Nrf2 was conducted in 183 patients with confirmed stage I to VI HNSCC. A higher level of Nrf2 was associated with a poorer overall survival (median OS: 45.5 months versus 60 months). This is further validated through the Cancer Genome Atlas (TCGA) database. The OS for Nrf2^high^ versus Nrf2^low^ is 40 months versus 90 months, and disease-free survival (DFS) in the Nrf2^high^ group is 64 months compared with 100 months in the Nrf2^low^ group. Nrf2 expression was significantly higher in cisplatin-resistant and nonresponder patients than good responders. HO-1, the Nrf2-targeted gene, was also elevated in cisplatin-resistant HNNC patients. Knockdown of Nrf2 reversed the sphere-forming efficiency that marks the cancer stem cell characteristics in FaDu cells [157]. Inhibition of Nrf2 by artesunate leading to a reversal of the ferroptosis resistance in cisplatin-resistant HNC cells has been reported [158]. These findings hint at some clues for the targeted therapy of the Nrf2/Keap1 system and complimentary strategy towards drug resistance.

### 4.6. Targeting the ETC Complexes in HNC

In cancer cells, mitochondria electron transporter chain (ETC) complexes become more active to produce ATP and ROS which induce drug resistance via ATP-driven multidrug efflux pumps. Elevated ROS promote certain antioxidant systems to attain redox balance. Therefore, disturbing ETC complexes show great potential for tackling drug resistance. On one hand is the consumption of as much ATP as possible, while on the other hand ROS levels are increased facilitating cellular apoptosis [40]. Proteomic expression profiling reveals reduction of COX7A2 (cytochrome *c* oxidase subunit 7A2), a subunit of ETC complex IV, which is related to patients with esophageal adenocarcinoma who respond to cisplatin plus 5-Fu therapy. Silencing of COX7A2 in OE19 cells leads to an abnormal cup-shaped structure of the mitochondria observed by electron microscopy. The combination treatment of cisplatin/5-Fu after silencing COX7A2 significantly inhibits OE19 cell proliferation [159].

## 5. Repurpose Old Drugs Modulating ROS for a New Life

The so-called “new use of old drugs” refers to the non-anti-cancer drugs that have been used for a long time in clinical practice. These drugs are applied to new fields because of their anticancer effects. By this, not only is the safety of drugs ensured, but the long cycle of new drug development and screening is also avoided (Table 2).

### 5.1. Sulfasalazine

Sulfasalazine is an anti-inflammatory drug that has been applied to treat inflammatory bowel disease and rheumatoid arthritis for decades [160]. Recent studies show that sulfasalazine, a nonsubstrate xCT inhibitor, can efficiently kill cancer cells. Sulfasalazine can eliminate cellular detoxification by GSH depletion and enhance the anticancer effect by upregulating ferroptosis in HNC [161]. In HNC cisplatin-resistant HN3-*cis*R, HN4-*cis*R, and HN9-*cis*R cells, 1 mM sulfasalazine can enhance cisplatin-induced cell death in terms of a significant decrease of GSH. Pretreatment of N-acetylcysteine (NAC) can block this effect. In HN9-*cis*R xenograft nude mice, a combination of sulfasalazine with cisplatin showed greater inhibition of tumor growth than either single group [162]. Thus, the synergy of sulfasalazine with conventional chemotherapeutic agents is promising in the treatment of advanced and resistant HNC.

### 5.2. Dichloroacetic Acid

Dichloroacetic acid (DCA), an inhibitor of pyruvate dehydrogenase kinase, has been approved by the FDA for treating a rare hereditary lactate metabolism disorder [163]. During the past decade, DCA has been repurposed for enhancing cancer therapy efficacy by overcoming resistance to chemotherapeutic drugs [164]. Even so, DCA has rarely been checked in HNC. Downregulation of PDK2 by DCA switches bioenergetics towards mitochondrial oxidative phosphorylation which leads to an increase in mitochondrial reactive oxygen species (mROS) in the larynx cancer cisplatin-resistant cell lines AMC-HN4-*cis*R and HN9-*cis*R, thus sensitizing a cisplatin effect in vitro and in vivo [165]. DCA-induced apoptosis by the inhibition of PDK1 in HNSCC cells can be further enhanced by cetuximab-mediated downregulation of ASCT2, which is a glutamine transporter [166]. One issue should be dealt with caution when the use of DCA in cancer treatment is concerned. Long-term exposure to DCA may shift normal cells such as immune cells to a greater oxidative metabolism in which the condition of normal physiology function is disturbed [164].

### 5.3. Melatonin

Melatonin, N-acetyl-5-methoxytryptamine, is a compound containing an indole ring approved by FDA as a raw material for dietary supplements. In China, the use of melatonin as a raw material for health foods is allowed, requiring a purity of more than 99.5% and a recommended daily dosage of 1-3 mg limited to improving sleep (product standard: GB/T5009.170-2003; http://www.nhc.gov.cn). During recent years, melatonin has been found to possess anti-inflammatory, antioxidant, and anticancer activities [167–171]. The melatonin gel [172] has completed a phase II clinical trial (EudraCT number: 2015-001534-13) in 80 patients with HNC. The results showed that melatonin can protect oral mucosa against the side effects of radiotherapy. Fernandez-Gil et al. have researched an enhancing cytotoxic role of melatonin combined with rapamycin in HNSCC cells. Moreover, they found that a high concentration of melatonin could sensitize HNC cells to CDDP and irradiation by enhancing the mitochondrial ROS and then inducing apoptosis and lethal autophagy [173]. A combined melatonin and irradiation treatment decreased the mitophagic marker NIX, while a combined melatonin and cisplatin treatment increased NIX [173]. This is perhaps due to different ROS levels enhanced by each kind of combination. Even so, melatonin shows great hope in combination with radiotherapy or chemotherapy for better therapeutic efficiency.

### 5.4. Thioridazine

Thioridazine was approved for use in the United States in 1978 and was indicated for the therapy of acute and chronic psychosis. A high concentration of thioridazine administration is prone to cause prolongation of the QTc interval and increase sudden death risk [174–176]. However, a low concentration of thioridazine is reported to induce apoptosis, inhibit angiogenesis and metastasis, and overcome drug resistance in cancer treatment [177–179]. A combination of thioridazine with carboplatin significantly induced mitochondrial apoptosis and downregulated apoptosis-related proteins c-FLIP and Mcl-2 which can be reversed by knockdown of PSMA5, a proteasome subunit. Besides, a combined treatment with carboplatin and thioridazine could induce ROS production and activate Nrf2 translocation as well as antioxidant response elements within 1 h in HNC AMC-HN4 cells. ROS scavengers (NAC, trolox, and glutathione-ethyl-ester) inhibited Nrf2 translocation and PSMA5 expression. Mitochondrial ROS have a critical role in carboplatin plus thioridazine-induced apoptosis. Moreover, a combination of thioridazine and carboplatin did not induce cell death in normal human mesangial and umbilical vein cells. Thus, a low concentration of thioridazine is a promising adjuvant agent in carboplatin-resistant HNC [180].

### 5.5. Acetylsalicylic Acid

Acetylsalicylic acid (aspirin), a nonsteroidal anti-inflammatory drug, has been used for relieving inflammation and preventing cardiovascular events [181]. It is reported that aspirin can inhibit tumor growth and metastasis [182]. A low concentration of aspirin (1-3 mM) can synergize 1-3 *μ*M sorafenib to more cell death in HNC cisplatin-resistant HN3R, HN4R, and HN9R cells. The combination of aspirin and sorafenib significantly decreased GSH level and elevated total ROS levels in cisplatin-resistant HNC cells. This effect can be revered by the antioxidant trolox. Furthermore, aspirin and sorafenib could synergize cisplatin-induced cytotoxicity in resistant HNC cells. In HN9R xenograft nude mice, the effect of aspirin plus sorafenib on cisplatin has been confirmed and that this trip-combination greatly suppressed tumor growth without affecting the weight of mice. Aspirin is promising in synergizing sorafenib alone or combined with sorafenib to synergize cisplatin in anticancer therapeutics of HNC [183].

### 5.6. Salinomycin

Salinomycin is a carboxypolyether potassium ionophore antibiotic isolated from the fermentation broth of *Streptomyces albus* by Miyazaki et al. in the process of screening for new antibiotics in 1974 [184]. Salinomycin has been widely used in the prevention and control of coccidiosis in poultry animals in the past. In 2009, Gupta et al. conducted a high-throughput screening of more than 16,000 chemicals and found that salinomycin can selectively kill breast cancer stem cells, and its killing effect is 100 times more than that of the clinical first-line chemotherapy drug paclitaxel [185]. The nasopharyngeal carcinoma radioresistant SUNE1R cells expressed higher Nrf2 compared to parental SUNE1 cells. Salinomycin can restore the radiosensitivity of SUNE1R cells by inducing apoptosis which is mediated via Nrf2 inhibition and ROS generation [186]. In view of these effects, salinomycin is perhaps a promising adjuvant agent to modulate ROS for enhancing the radiosensitivity of HNC. However, more in vivo experiments concerning its efficacy and toxicity should be further carried out.

### 5.7. Metformin

Metformin has been approved to treat type 2 diabetes since 1957 in Europe [187]. Due to lactic acidosis, metformin was taken off the US market; however, later it has been proven safe and effective in controlling glucose levels and was reapplied in 1995 [188, 189]. In 2005, metformin was used to reduce the incidence of cancer in patients with diabetes [190]. Since then, metformin has been vastly explored in the anticancer field. Metformin has been reported to inhibit the proliferation and viability of HNSCC cells via an AMPK-dependent manner [191]. In another research, metformin could suppress both HNSCC HN30 (wtp53) and HN31 (p53 with 2 missense mutations) cells via the downregulation of malic enzyme 2 (ME2) driven by ROS generation. Noteworthy, metformin exerted a more efficient inhibitory effect in HN31 cells which are resistant to radiation [192]. This provided an AMPK-independent manner for metformin to enhance the radiation effect against resistant HNSCC.

## 6. Exploit Novel Small Molecular Compounds Targeting ROS

Small molecular compounds composed of several or dozens of atoms have always been commonly used in clinical medicine due to their many advantages, such as a definite curative effect, less adverse effects, and smaller molecular weight, which are easily absorbed [193]. It is also one of the hot spots in the field of medicinal chemistry drug development. Based on the intensive implication of ROS in cancer treatment, here we reviewed several novel compounds modulating ROS as a potential adjuvant therapy of HNC (Table 3).

### 6.1. CHW09

Chromones are oxygen-containing heterocyclic compounds that possess anti-inflammatory and anticancer abilities. A sulfonyl substituent is installed on the chrome-4-one skeleton. This synthesized compound is named CHW09. In vitro, CHW09 can efficiently kill oral cancer cells Ca9-22 and CAL 27 with a mild decrease in viability in the normal human gingival fibroblast cell HGF-1. Cellular ROS and mitochondrial superoxide were both induced, and subsequent apoptosis and DNA damage were enhanced after the treatment of CHW09. The high-stress status renders cancer cells more sensitive to ROS-generating agents [194]. A combination of 10 *μ*g/ml CHW09 and 12 Gy radiation synergistically inhibits proliferation and induces apoptosis of Ca9-22 and CAL 27 [195]. However, the animal experiments are not available now.

### 6.2. Oxamate

Lactate dehydrogenase (LDH) is a major glycolytic enzyme which catalyzes the transformation of pyruvate to lactate. As the Warburg effect commonly exists in cancer cells with elevated glucose consumption and aerobic glycolysis, the LDH expression is increased at the same time in various types of cancer [196]. Oxamate, a LDH competitive inhibitor, provides an attractive chance to develop a novel cancer therapeutic strategy. In nasopharyngeal carcinoma CNE-1 and CNE-2 cells, oxamate efficiently synergizes radiation by upregulating ROS level and subsequent G_2_/M arrest and apoptosis. Besides, inhibition of LDH disturbed energy metabolism and significantly decreased ATP production. An in vivo experiment further validated the synergizing effect of oxamate in radiation [197]. Even so, the small size and high polarity of oxamate limit its catalytic activity and permeability. Several N-alkyl-oxamates are synthesized [198]. Further experiments are imperative concerning their inhibitory effects on LDH and anticancer actions.

### 6.3. D-Allose

D-Allose is a rare aldohexose with many physiological functions including lowering blood lipid and blood glucose concentrations, scavenging free radicals in the body, and reducing ischemia-reperfusion injury and anticancer effects [199]. It is noteworthy that D-allose can inhibit carcinogenesis under oxidative stress and can induce the expression of TXNIP which inhibits the proliferation of HNC cells [200]. Hoshikawa et al. reported the radiosensitizing effect of D-allose on HNC HSC-3 cells using a 3D culture method. A combination of D-allose and radiotherapy had better effects than the two alone. The radiation enhancement rate reached 1.61 and 2.11 after 10 mM and 25 mM allose treatment, respectively. The radiation treatment alone could not increase the expression of the mRNA level of TXNIP, while allose combined with radiation treatment could significantly increase the expression of TXNIP which can significantly induce the generation of cellular ROS and the occurrence of apoptosis [201]. Besides, D-allose can synergize docetaxel-induced apoptosis by increasing TXNIP and ROS in vitro and in vivo [202]. Most importantly, allose has no known side effects [203], so the combined use of allose and radiation or docetaxel may become a new treatment strategy for HNC [204].

### 6.4. Histone Deacetylase Inhibitors

Tumorigenesis and progression are the result of the interaction of heredity and epigenetics. As an important epigenetic modification, histone deacetylation plays an important role in the occurrence and development of a tumor [205]. The abnormal expression of histone deacetylase (HDAC) in normal tissues and cells will promote the development of a tumor, and it is related to the proliferation and apoptosis, angiogenesis, metastasis, and drug resistance of tumor cells, and becomes a new target of tumor treatment [206–209]. HDAC inhibitors such as vorinostat (SAHA), romidepsin, belinostat, and panobinostat have been approved by FDA as anticancer drugs (https://www.fda.gov). More combination modalities concerning SAHA with conventional chemotherapy drugs are undergoing preclinical researches [146, 209, 210]. SAHA can synergize bortezomib-induced apoptosis via the upregulation of ROS in nasopharyngeal carcinoma cells. Further in vivo experiments confirmed this effect [146]. Sodium butyrate (NaB) and hydroxamic acid trichostatin A (TSA) are another two HDAC inhibitors that sensitize radiation by downregulating Bmi-1 and then increasing ROS generation and impairing DNA repair in esophageal squamous cell carcinoma radioresistant KYSE-150R cells. HDAC inhibitors as anticancer drugs complementary to chemo-/radiotherapy show a great potential [211].

## 7. Natural Herbs Effectively Modulating ROS Are Important Drug Candidates

Natural herbs combined with surgery and chemo-/radiotherapy show a certain effect in clinical cancer treatment. Mechanistically, the imbalance between ROS generation and elimination in cancer provides an opportunity for natural herbs. Generally, ROS upregulation synergizes conventional chemo-/radiotherapy, while the downregulation of ROS may protect normal tissue from side effects. Here, we reviewed several natural herbs modulating ROS in the comprehensive treatment of HNC (Table 4).

### 7.1. Flavonoids

Flavonoids, a group of important naturally occurring compounds found in several edible vegetables, fruits, and medicinal plants, are structured by connecting two benzene rings with phenolic hydroxyl groups through the central three-carbon chain (C_6_-C_3_-C_6_) [212]. It is reported that flavonoids can be used to protect the cardiovascular system, lower diabetes risk, cure neurodegenerative disorders, restore cognition after stroke, and suppress cancer progression [213–215]. Although flavonoids do not seem to be potent enough to be used as a monotherapy in the treatment of cancers, these compounds have been suggested to render considerable clinical benefits when applied in combination with radiotherapy or chemotherapy. Quercetin can synergize cisplatin-induced mitochondrial apoptosis via downregulating Cu/Zn SOD which leads to elevated ROS in larynx cancer Hep2 cells [216]. Naringin has a protective role in doxorubicin-induced toxicity towards normal tissues without sacrificing its anticancer effect by elevating SOD and total antioxidant capacity against the esophageal cancer stem cell YM1 in xenograft nude mice [217]. Alpinumisoflavone (AIF) could significantly increase the radiosensitivity of esophageal squamous cell carcinoma (ESCC) indicated by enhanced apoptosis, DNA damage, and cell cycle arrest which are mechanically achieved by ROS generation and Nrf2 antioxidant system inhibition both in vitro and in vivo [218]. Wogonin, isolated from the root of *Scutellaria baicalensis Georgi*, could selectively kill HNC cells by upregulating intracellular ROS with no obvious cytotoxic effect against normal oral keratinocytes, oral fibroblasts, and skin keratinocytes. Mechanically, wogonin induces HNC cell death via JNK and PARP activation resulting from the inhibition of Nrf2-GSTP1. Combined wogonin synergizes cisplatin-induced cell death of cisplatin-resistant HNC HN4R and HN9R cells by enhanced ROS in vitro and in vivo. These findings show great hope in the chemosensitivity potential of wogonin in advanced HNC [219].

### 7.2. Polyphenols

Curcumin is a hydrophobic phenol isolated from *Curcuma longa* and possesses a variety of pharmacological effects including antidiabetic, antiamyloid, antidepressant, antibacterial, cardioprotective, anti-inflammatory, antioxidant, and anticancer properties [220]. Multiple animal and human studies prove that curcumin is nontoxic even at high doses [221]. Curcumin can inhibit the effects of prosurvival and antiapoptotic elements such as NF-*κ*B and reduce the radiation adaptation in order to enhance the radiation-induced killing effect in various cancer cells [222]. A higher expression of TxnRd1 leads to intrinsic resistance to radiation in HPV^−^ cells. Curcumin can effectively downregulate TxnRd1 and then sensitize HPV^−^ cells to radiation [145]. In a very recent research, curcumin and ferulic acid (FA) both show an antioxidant ability by upregulating the Nrf2/HO-1 pathway for protecting the cochlea after cisplatin treatment without sacrificing the anticancer therapeutic effect in the human oral squamous carcinoma cell line PE/CA-PJ15 and in animal models. One thing to mention is that FA exhibits a biphasic response wherein at lower concentrations it exerts an oxidant function and at higher concentrations it promotes an antioxidant function for chemoresistance. Judging from this, curcumin seems the optimum regimen for effective treatment [223]. A novel synthetic polyphenol conjugate, (E)-3-(3,5-dimethoxyphenyl)-1-(2-methoxyphenyl) prop-2-en-1-one (DPP-23), can efficiently kill cisplatin-resistant HN3-*cis*R, HN4-*cis*R, and HN9-*cis*R cells without harming normal HOK-1 cells. DPP-23 inhibits Nrf2 antioxidant systems and activates p53 expression, thus boosting an increase in cisplatin-mediated apoptosis in vitro and in vivo [224]. Epigallocatechin gallate (EGCG) and tannic acid (TA) could mitigate doxorubicin-induced keratinocyte toxicity without impairing the anticancer effect of doxorubicin at a certain concentrations. An additive cellular ROS increase was not observed after combination treatment of doxorubicin with either 50 *μ*M EGCG or 50 *μ*M TA in oral keratinocyte cells [225]. Epicatechin can protect normal oral fibroblasts from radiation via downregulating ROS and subsequent apoptosis. This is also validated in zebrafish. Epicatechin inhibits JNK and p38 signaling pathways but not the ERK pathway during this physiological process [226]. Another study also confirmed a radioprotective role of epicatechin in human keratinocyte HaCaT cells and in Sprague-Dawley rats via ROS regulation and JNK and p38 pathway alterations [227].

### 7.3. Naphthoquinones

*β*-Lapachone (3,4-dihydro-2,2-dimethyl-2H-naphthol (1,2-b) pyran-5,6-dione (C_15_H_14_O_3_)) is a natural naphthoquinone, originally an isomer of lapacho, obtained from the bark of the *purple Ipe* in South America. Various studies have demonstrated that *β*-lapachone can induce cell death in solid cancers including esophageal and oral cancers [228–230]. ARQ 761, a *β*-lapachone analogue, has completed a phase I clinical trial (clinical trial number: NCT01502800) in advanced solid tumors. Outcomes show that ARQ 761 possesses a modest single-agent activity. The most common adverse effect is anemia [231]. Several derivatives have been developed throughout the years. *β*-Lapachone and its 3-iodine derivatives (3-I-*α*-lapachone and 3-I-*β*-lapachone) efficiently kill OSCC HSC-3 cells by enhancing ROS and inducing G_2_/M arrest, DNA fragmentation, and mitochondria-dependent apoptosis. These results are synchronized in an in vivo study, and the toxicology towards normal tissue is slight [232]. In another multifaceted study, NQO1 is highly expressed in HNC clinical tissue samples, and *β*-lapachone can synergize radiation to enhance apoptosis and DNA damage by inhibiting NQO1 in HNC FaDu, Detroit 562, SqCC/Y1, and UMSCC-10A cells and also in SqCC/Y1 xenograft nude mice [233]. Thus, the combination treatment of *β*-lapachone and radiotherapy for QNO1^+^ HNC patients shall be further tested in clinical trials.

Plumbagin (5-hydroxy-2-methyl-1, 4-naphthoquinone (C_11_H_8_O_3_)), isolated from *Plumbago zeylanica* L., *Juglans regia*, *Juglans cinerea*, and *Juglans nigra*, exerts antibacterial, antifungal, antiatherosclerosis, and anticancer effects [234]. Our research group has devoted to research its anticancer properties in HNC in recent years. Plumbagin can induce ROS, G_2_/M arrest, apoptosis, and autophagy in addition to reversing Epithelial-Mesenchymal Transitions (EMT) and cancer stem cell characteristics via inhibiting PI3K/Akt/mTOR, GLUT-1, MAPK, and Nrf2 signaling pathways of oral squamous cell carcinoma (OSCC) in vitro and in vivo [235–237]. In our very recent research, the cisplatin-resistant cell line CAL27/CDDP is applied to verify the chemosensitivity of plumbagin in cisplatin treatment. Outcomes show that plumbagin can efficiently synergize cisplatin-induced apoptosis via upregulating cellular ROS and mitochondrial hydrogen peroxide. Autophagy is also induced by plumbagin and cisplatin, while it is hard to determine its definite anticancer or protective role. Besides, these effects can all be reversed by antioxidant NAC. In CAL27/CDDP xenograft nude mice, we are glad to observe that the combination of plumbagin and cisplatin can significantly reduce tumor volume without affecting the weight of the mice [238]. In order to prompt the clinical utility of plumbagin, we also carried out stable isotope labeling with amino acids in cell culture (SILAC) quantitative proteomics technology to fully reveal the possible molecular targets of plumbagin on OSCC [236]. More well-designed experiments are going on to determine plumbagin's anticancer effect in chemo-/radiotherapy in HNC.

### 7.4. Terpenoids

Oridonin is a natural bioactive diterpenoid isolated from *Rabdosia rubescens*, which has been a widely used herb in traditional Chinese medicine [239]. Oridonin shows great anticancer potential with low adverse effect [240]. In human laryngeal squamous cell carcinoma (LSCC) Hep-2 cells, oridonin can induce G_2_/M phase arrest and apoptosis by targeting caspase 9 to enhance ROS production [240, 241]. Hep-2 is a cell line characterized by high EGFR expression. Hence, the inhibition of EGFR with tyrphostin AG1478 can augment oridonin-induced intrinsic and extrinsic apoptosis via ROS generation in Hep-2 cells [242]. Oridonin is reported to synergize cetuximab. The setting is that cetuximab exhibits unsatisfactory efficacy as a single agent in HNSCC patients [243]. The combined treatment with oridonin and cetuximab could induce Fas-dependent apoptosis and G_2_/M arrest through triggering ROS generation in LSCC Hep-2 and Tu212 cells. EGFR and JNK signaling pathways are involved in these biological effects. In vivo experiments validate the combined anticancer effect of oridonin and cetuximab [244]. Thus, oridonin is a promising drug targeting ROS in combination with cetuximab in resistant cases.

### 7.5. Ginsenosides

Ginsenosides, the main active ingredient of ginseng, have significant anticancer activity by inhibiting cell proliferation, promoting apoptosis, inducing cell cycle arrest of cancer cells, inhibiting tumor angiogenesis, and synergizing with chemo-/radiotherapy [245]. Besides, ginsenosides can activate the body's immunity through different ways to fight against cancer [246]. Some kinds of ginsenosides are undergoing clinical trials [247]. Ro, a kind of ginsenoside monomer, can activate estrogen receptor 2 (ESR2), which leads to the activation of neutrophil cytosolic factor 1 (NCF1), a subunit of NADPH oxidase, and then leads to the elevation of ROS production. It is reported that Ro can synergize the killing effect of 5-fluorouracilin by upregulating ROS and subsequently inhibiting protective autophagy in esophageal cancer ECA-109 and TE-1 cells. NAC, an antioxidant, substantially reversed Ro-mediated autophagy inhibition in ECA-109 and TE-1 cells and reversed cell death enhanced by the combination of Ro and 5-fluorouracilin [248]. Korean red ginseng (KRG) whose effective constituent is ginsenoside shows great potential in radiosensitivity in oral cancer SCC25 and SCC1484 cells and radioprotection in normal keratinocyte HaCaT cells. Radiation can induce cell death in HaCaT cells by increasing intracellular ROS and membrane damage. When radiation is combined with KRG, the injury of HaCaT cells was greatly alleviated accompanied by ROS elimination and downregulation of p38 and JNK signaling pathways. This protective effect was verified in a zebrafish embryo toxicity model. These findings show that KRG can potentially be used as a protective drug against radiation-induced oral mucositis without impairing the killing effect of cancer cells [249].

## 8. Emerging Interdisciplinary Techniques Broaden the Potency of ROS in Chemo-/Radiotherapy in HNC

### 8.1. Application of Photodynamic Therapy (PDT)

Photodynamic therapy (PDT) is a recognized treatment for incurable head and neck cancer [250, 251]. PDT may be particularly useful for the treatment of early unresectable lesions and remission of locally recurrent esophageal cancer [252], resulting in prolonged survival [253]. Besides, the application of PDT will not affect treatment options for future relapses or second primary disease [254]. Conventional PDT starts with the administration of a photosensitizer (PS), which is excited by locally applied light after 2-4 days. The activated PS subsequently converts oxygen to ROS that can damage DNA, proteins, and lipids, ultimately resulting in cell death [255]. However, side effects of conventional PDT (using hydrophobic PS) are common, including damage to normal surrounding tissues and skin phototoxicity. Hence, there is a trend that associates PDT with other chemotherapeutic agents to reduce tumor resistance and improve the efficacy of treatment [256]. Targeted PDT with cetuximab-IRDye700DX conjugates is currently being tested in patients diagnosed with advanced stage HNSCC (clinical trial number: NCT02422979). The first results of this trial indicate that patients responded well to this therapy, while experiencing limited side effects [257]. The following table lists some recent researches concerning combinations of PDT with chemotherapy drugs which show a synergistic anticancer effect and are expected in future clinical trials (Table 5).

### 8.2. Application of the Nanoparticle System Based on ROS Modulation

Nanomedicine is an emerging form of treatment that is dedicated to alternative drug delivery and improved therapeutic efficacy, while reducing harmful side effects on normal tissues. New nanoparticle systems with highly flexible and rapid drug design and production capabilities can be created, which can be designed based on the genetic characteristics of tumors; therefore, making the drug selection for individual patient treatment and overcoming multiple forms of multidrug resistance look promising and open up new prospects for cancer treatment [262–268]. The effect of metal nanoparticles on ROS concentration has been shown to play a role in both radiosensitization and radioprotection. Many studies have investigated the effects of nanoparticle structure and surface functionalization on nanoparticle absorption and radiosensitization during radiotherapy [269].

In OSCC cells, RPTD/HP nanoparticles were effectively internalized and showed effective effects on cell growth inhibition and apoptosis induction after laser irradiation. In OSCC tumor-bearing mice, RPTD/HP nanoparticles show excellent tumor-targeting ability and significantly inhibit tumor growth through a variety of mechanisms after local laser irradiation [270].

Cur-NPs induce apoptosis and inhibit cell growth in human oral cancer cisplatin-resistant CAL27 cells, but they have no cytotoxicity on normal human gingival fibroblast cells and normal keratinocyte cells. The results show that Cur-NPs trigger apoptotic cell death by regulating the function of MDR1 and the production of ROS. The activation of caspase 9 and caspase 3 associated with intrinsic signaling pathways is its main pharmacological effect. Cur-NPs are expected to become a new drug against cisplatin-resistant human oral cancer [271].

### 8.3. BEMER Therapy Possesses Great Potential in Radiosensitization

Application of low-dose electromagnetic fields (EMF) in the regulation of cellular processes is a complementary therapeutic method. EMF therapy can effectively normalize the tissue microcirculation, thus sensitizing cancer therapy [272–277]. A unique system is the Bio-Electro-Magnetic-Energy-Regulation (BEMER) which employs a low-frequency pulsed magnetic field (maximum 35 *μ*T) with a series of half-wave-like sinusoidal intensity transformation. The BEMER system facilitates an increase in blood vessel and microcirculation to improve organ blood flow, nutrient supply, and removal of metabolites. BEMER treatment showed an obvious radiosensitizing effect in a time-dependent manner by deriving a high ROS level and increasing the number of DNA double-strand breaks (DSBs) in the HNC cell line UTSCC15 which were cultured in a 3D laminin-rich extracellular matrix [278].

## 9. Conclusions and Perspectives

The estimated new cases of HNC have gradually increased during the past ten years [7, 13, 279]. The five-year survival rate of HNC has not been significantly improved especially in esophageal cancer which is as low as 12% [3]. Two-thirds of the patients with HNC are advanced cases when they are first examined [20]. Comprehensive modality is required for advanced HNC covering surgery, radiotherapy, and chemotherapy. However, response failures and severe side effects still affect the prognosis and quality of life of patients. Methods that simultaneously increase the therapeutic response of cancer cells and protect normal tissues are needed to ameliorate the treatment outcome.

The cellular redox status greatly affects the chemo-/radiotherapy efficiency directly or indirectly by multiple biological events such as cell death induction [45–47], DNA damage repair [45–47], stemness maintenance [68], metabolic reprogramming [81, 82], and tumor microenvironment modification [78]. In the past decade, researchers around the world have carried out numerous researches dedicated to enhancing and adjuvanting the effect of chemo-/radiotherapy by finely adjusting the cellular redox of HNC. These research findings contribute to extend the molecular mechanisms and orchestrate therapeutic strategies to overcome resistance and reduce the side effects, and finally improve long-term outcomes for HNC.

Increasing efficacy may be obtained by combining agents with established conventional treatments. In this review paper, we list several “old drugs” and novel small molecular compounds which efficiently modulate cellular or mitochondrial ROS levels to synergize chemo-/radiotherapy. The mechanisms in terms of energy metabolism, cell death, and DNA damage are investigated. Redox-related signaling pathways such as Nrf2/Keap1, MAPKs, p53, NF-*κ*B, and STAT3 have been discovered to be involved in the process of chemo-/radiosensitization at different degrees using multiple cell and animal models.

In view of the dual role of ROS at different cell stages and the fact that the basic level is different for different types of cells, it should require considerable caution when adjusting ROS. In general, the success of cancer treatments by inducing oxidative damage or disrupting antioxidant systems to suspend the cancer progression of redox homeostasis should be tailored according to tumor stage and pathological pattern, antioxidant levels in the microenvironment of the tumor, and the endogenous antioxidant capacity [6]. Oxidation-reduction screening may include the expression rates of different oxidoreductase enzymes and the comparison of antioxidant enzyme expression between tumor tissues and normal tissues [280]. Prominent detection kits by simple sampling methods concerning the redox status of patients with HNC may bring benefits. This approach can be used to better scheme the treatment for each patient and maximize the effectiveness of the treatment to annihilate the cancerous tissue and reduce adverse harm to normal tissue.

Besides, the overexpression of EGFR was discovered in 80-100% of HNC patients [281]. Inhibition of EGFR strengthens the apoptosis induction of ROS-generating agents in HNC. In the context of EGFR, the downregulation of the glutamine transporter ASCT2 can sensitize HNSCC cells to combination therapy with radiation, cetuximab, or cisplatin to induce higher ROS and then evoke more apoptosis [166, 282]. Ptoxin, a new immunotoxin, was obtained by fusing a novel EGFR-targeted antibody into pseudomonas exotoxin A. Ptoxin can effectively boost ROS via inhibiting the Nrf2-Keap1 antioxidant pathway, thus inducing apoptosis in EGFR^+^ esophageal cancer cells [283]. DpdtbA, a dithiocarbamate derivative, can effectively inhibit p53/EGFR/AK and produce ROS via inactivating and downregulating SOD which lead to the occurrence of apoptosis [284]. In the future, the combination of Ptoxin and/or DpdtbA with chemotherapy or radiotherapy shows great potential against the most deadly esophageal cancer.

Noteworthy is the emergence of natural herbs that are considered to be putative chemo-/radiosensitizers. Due to the drug resistance of small molecule-targeted inhibitors, researchers are currently committed to developing “double target” or “multitarget” agents. The value of natural herbs is their capability of exerting their anticancer ability via multiple targets which may be developed as effective and ideal drugs to improve cancer treatment especially under the complex circumstance of redox. Defects such as poor solution, low bioavailability, and inefficient extraction of natural herbs limit their future application. Several approaches including the development of synthetic analogues, the use of nanoparticles and other efficient delivery agents to improve bioavailability, and the employment of phospholipid complexes to increase solubility have shown promise in overcoming these challenges [285, 286]. Interdisciplinary techniques such as PDT, nanoparticle transfer system, and the BEMER system show great potential in personalized ROS modulation at a large scale in future combination therapy. More mechanistic studies and randomized controlled trials are required to confirm the benefits.

## Figures and Tables

**Table 1:** The advantages and disadvantages of several ROS probes.

Name	Advantages	Disadvantages	Reference
DCFH-DA	Convenient to use	Photosensitivity and autoxidation; not specified to detect H_2_O_2_; oxidized by cytochrome *c*	[86, 87]
DHE	Convenient to use; specified to detect O_2_^−^	Produces two products with similar fluorescence characteristics which need to be resolved by HPLC and other means; photosensitivity and autoxidation	[88]
DHR	Convenient to use; specified to detect ONOO^−^	Intermediates can be reduced by mercaptan and vitamin C; autoxidation	[89]
FlAmBE	Convenient to use; stable fluorescence	Not specified to detect ONOO^−^; high background fluorescence	[90]
HKSOX-1/1r	Specified to detect superoxide; stable fluorescence; specified to detect O_2_^−^; insensitive to low pH	Not clear	[91]
MitoSOX	TPP group localized in mitochondria; convenient to use; specified to detect O2^−^	Interferes with mitochondrial metabolism; mitochondrial membrane; potential-dependent location; produces two products with similar fluorescence characteristics which need to be resolved by HPLC; photosensitivity and autoxidation	[92]
MitoPY1	TPP group localized in mitochondria; convenient to use; stable fluorescence	Mitochondrial membrane potential-dependent location; not specified to detect ONOO^−^; high background fluorescence	[93]
MitoAR/HR	Rhodamine group localized in mitochondria; convenient to use; specified to detect ·OH/HClO	Mitochondrial membrane potential-dependent location	[94]
HKSOX-1m	TPP group localized in mitochondria; specified to detect O_2_^−^; stable fluorescence; insensitive to low pH	Mitochondrial membrane potential-dependent location	[91]
FRR2	Rhodamine group localized in mitochondria; convenient to use; reversible real-time detection; stable fluorescence	Nonspecific; mitochondrial membrane potential-dependent location	[95]
Pep1-NP	Cationic styrene localized in mitochondria; convenient to use; specified to detect H_2_O_2_; stable fluorescence	Not clear	[96]
Hyper	Highly specific to H_2_O_2_; reversible real-time detection; stable fluorescence; MLS group localized in subcellular structure; independent of membrane potential	pH sensitive; limitation of cell transfection efficiency	[97]
RoGFP2-Orp1	Highly specific to H_2_O_2_; reversible real-time detection; stable fluorescence; MLS group localized in subcellular structure; independent of membrane potential; pH insensitivity	Limitation of cell transfection efficiency	[98]

*Note*. DCFH-DA: 2,′7′-dichlorofluorescein diacetate; H_2_O_2_: hydrogen peroxide; DHE: dihydroethidium; O_2_^−^: superoxide anion radical; DHR: dihydrorhodamine; ONOO-: peroxynitrite anion; FlAmBE: boric acid ester derivative; HKSOX-1/1r/1m: novel O_2_- probes using carboxy tetrafluorofluorescein as fluorescence group (HKSOX-1/1r for cellular retention, HKSOX-1m for mitochondria-targeting); pH: potential of hydrogen; MitoSOX: DHE for mitochondria-targeting; TPP: triphenyl-phosphine; HPLC: high-performance liquid chromatography; MitoPY1: FlAmBE for mitochondria-targeting; MitoAR/HR: DHR for mitochondria-targeting; ·OH: hydroxyl radical; HClO: hypochlorous acid; FRR2: a novel DHR probe; Pep1-NP: a novel boric acid probe targeting mitochondria; Hyper: a genetic probe specific for H_2_O_2_; RoGFP2-Orp1: redox-sensitive green fluorescent proteins 2; MLS: mitochondrial localization sequences.

**Table 2:** Old drugs modulating ROS as an adjuvant agent in the chemo-/radiotherapy of HNC.

Drug	Site	Experimental model	Effective dose	Cotherapy	ROS detection	Biological effects	Mechanisms	Reference
Sulfasalazine	Larynx	In vitro (HN3, HN4, and HN9; HN3-*cis*R, HN4-*cis*R, and HN9-*cis*R cells) In vivo (HN9-*cis*R xenograft nude mice)	In vitro (1 mM) In vivo (250 mg/kg daily)	+Cisplatin In vitro (20 *μ*M) In vivo (5 mg/kg weekly)	DCFH-DA flow cytometry	Synergistic effect	↑ROS, ↓GSH, ↓xCT, ↑*γ*H2AX	[162]

DCA	Larynx	In vitro (HN2, 3, 4, 5, 9, and 10; SNU-1041, 1066, and 1076; HN4-*cis*R and HN9-*cis*R cells) In vivo (HN4-*cis*R and HN9-*cis*R xenograft nude mice)	In vitro (15-30 mM) In vivo (0.5 g/l once per week)	+Cisplatin In vitro (10-30 *μ*M) In vivo (5 mg/kg once per week)	DCFH-DA+MitoSOX flow cytometry and confocal microscopy	Synergistic effect: enhances apoptosis	↑mROS, ↓*ΔΨ*m, ↓PDK2, ↑p21, ↓pPDHE1*α*, ↑c-PARP, ↑PUMA, ↑CC3	[165]

Melatonin	Oral cavity	In vitro (Cal-27, SCC-9 cell)	1.5 mM	+Radiation (8 Gy)	DCFH-DA spectrofluorometer	Synergistic effects: enhance apoptosis and lethal autophagy	↑GSSG/GSH, ↑Bax/Bcl-2, ↓NIX, ↑ATG12-ATG5	[173]

Melatonin	Oral cavity	In vitro (Cal-27, SCC-9 cell)	1.5 mM	+Cisplatin (10 *μ*M)	DCFH-DA spectrofluorometer	Synergistic effects: enhance apoptosis and lethal autophagy	↑GSSG/GSH, ↑Bax/Bcl-2, ↑NIX, ↑ATG12-ATG5	[173]

Thioridazine	Larynx	In vitro (AMC-HN4 cell)	10 *μ*M	+Carboplatin	DCFH-DA+MitoSOX flow cytometry and fluorescence microscope	Synergistic effect: enhances apoptosis	↑ROS, ↓PSMA5, ↑Nrf2, ↓c-FLIP, ↓Mcl-1, ↑c-PARP, ↑CC3	[180]

Aspirin	Larynx	In vitro (HN3, 4, and 9; HN3R, 4R, and 9R cells) In vivo (HN9R xenograft nude mice)	In vitro (5-10 mM) In vivo (10 mg/kg daily)	+Sorafenib In vitro (5-10 *μ*M) In vivo (10 mg/kg daily)	DCFH-DA flow cytometry	Synergistic effect	↑ROS, ↓xCT, ↓GSH, ↑c-PARP, ↓p65, ↓Mcl-1	[183]

Aspirin	Larynx	In vitro (HN3, 4, and 9; HN3R, 4R, and 9R cells) In vivo (HN9R xenograft nude mice)	In vitro (5-10 mM) In vivo (10 mg/kg daily)	+Cisplatin In vitro (10 *μ*M) In vivo (5 mg/kg weekly)	DCFH-DA flow cytometry	Synergistic effect	↓xCT, ↓GSH, ↑c-PARP, ↓p65, ↓Mcl-1, ↑p-p53	[183]

Salinomycin	Nasopharynx	In vitro (CNE-1, CNE-2, SUNE1, 6-10B, 5-8F, SUNE1R cell)	2 *μ*M	+Radiation (4 Gy)	DCFH-DA flow cytometry	Synergistic effect: enhances apoptosis	↑ROS, ↓Nrf2, ↓survivin	[186]
Metformin	HNSCC	In vitro (HN30, HN31 cell) Clinical samples	2.5 mM	+Radiation (4 Gy)	DCFH-DA flow cytometry	Synergistic effect: induces senescence	↑ROS, ↓ME2, ↑p21, ↑NADP/NADPH, ↑SA-*β*-gal	[192]

*Note*. mM: millimole; *μ*M: micromole; DCFH-DA: 2′,7′-dichlorofluorescein diacetate; ROS: reactive oxygen species; GSH: glutathione; GSSG: oxidized glutathione; xCT: cysteine-glutamate antiporter; *γ*H2AX: H2A histone family member X; DCA: dichloroacetic acid; mROS: mitochondrial reactive oxygen species; *ΔΨ*m: mitochondrial membrane potential; PDK2: pyruvate dehydrogenase kinase 2; p21: protein 21; PDHE1*α*: pyruvate dehydrogenase E1-*α*; c-PARP: cleaved poly-ADP ribose polymerase; PUMA: p53 upregulated modulator of apoptosis; CC3: cleaved caspase 3; Bcl-2: B-cell lymphoma-2; Bax: Bcl-2-associated X protein; NIX: adenovirus E1B 19 kDa interacting protein 3-like; ATG: autophagy related; PSMA5: proteasome subunit alpha 5; Nrf2: nuclear factor E2-related factor 2; c-FLIP: cellular FLICE-like inhibitory protein; Mcl-1: myeloid cell leukaemia-1; p65: protein 65; p-p53: phosphorylated protein 53; ME2: malic enzyme 2; NADP: nicotinamide adenine dinucleotide phosphate; NADPH: nicotinamide adenine dinucleotide phosphate oxidase; SA-*β*-gal: senescence-associated *β*-galactosidase.

**Table 3:** Small molecular compounds modulating ROS in chemo-/radiotherapy of HNC.

Compound	Site	Experimental model	Effective dose	Cotherapy	ROS detection	Biological effect	Mechanisms	Reference
CHW09	Oral cavity	In vitro (Ca9-22, CAL 27 cancer cell, and normal gingival fibroblast HGF-1 cell)	10 *μ*g/ml	+Radiation (12 Gy)	DCFH-DA flow cytometry	Synergistic effects	↑ROS, ↑CC3, ↑CC8, ↑c-PARP, ↑8-oxodG, ↑*γ*H2AX	[195]

Oxamate	Nasopharynx	In vitro (CNE-1, CNE-2 cell) In vivo (CNE-1 xenograft nude mice)	In vitro (20, 50, 100 mM) In vivo (750 mg/kg daily for 3 weeks)	+Radiation (9.9 Gy)	DCFH-DA flow cytometry	Synergistic effect: enhances apoptosis and G_2_/M arrest	↑ROS, ↓ATP, ↓CDK1/cyclin B1, ↓Bcl-2, ↑Bax, ↑CC3	[197]

D-Allose	Tongue	In vitro (HSC-3 cell)	25 mM	+Radiation (4 Gy)	DCFH-DA fluorescence microscopy	Synergistic effect: enhances apoptosis	↑ROS, ↑TXNIP, ↓TRX	[201]

D-Allose	Tongue	In vitro (HSC-3 cell) In vivo (HSC-3 xenograft nude mice)	In vitro (10 mM) In vivo (500 mM 5 times/week for 3 weeks)	+Docetaxel In vitro (0.1 ng/ml) In vivo (12 mg/kg on day 0 and day 7)	DCFH-DA fluorescence microscopy	Synergistic effect: enhances apoptosis G_2_/M arrest	↑ROS, ↑TXNIP, ↓TRX	[202]

SAHA	Nasopharynx	In vitro (HK1-EBV, HONE1-EBV, HA, C666-1, NP460, HK2 cell) In vivo (C666-1, HONE1, HA xenograft nude mice)	In vitro (5 *μ*M) In vivo (50 mg/kg 5 days per week for 4 weeks)	+Bortezomib In vitro (30 nM) In vivo (60 *μ*g/kg)	DCFH-DA flow cytometry	Synergistic effect: enhances apoptosis	↑ROS, ↑c-PARP, ↑CC3, ↑CC7, ↑CC9	[146]

NaB	Esophagus	In vitro (KYSE-150, KYSE-150R cells)	0.5, 1 *μ*M	+Radiation (5 Gy)	DCFH-DA flow cytometry	Synergistic effect: enhances apoptosis, G_2_/M arrest, and DNA damage	↑ROS, ↓Bmi-1, ↑p21, ↓DNA-PKcs, ↓NBS1, ↓Rad51, ↑*γ*H2AX	[65]

TSA	Esophagus	In vitro (KYSE-150, KYSE-150R cells)	5, 10 mM	+Radiation (5 Gy)	DCFH-DA flow cytometry	Synergistic effect enhances apoptosis, G_2_/M arrest, and DNA damage	↑ROS, ↓Bmi-1, ↑p21, ↓DNA-PKcs, ↓NBS1, ↓Rad51, ↑*γ*H2AX	[65]

*Note*. ROS: reactive oxygen species; DCFH-DA: 2′,7′-dichlorofluorescein diacetate; CHW09: sulfonyl chromen-4-ones; SAHA: vorinostat; NaB: sodium butyrate; TSA: hydroxamic acid trichostatin A; c-PARP: poly-ADP ribose polymerase; CC3: cleaved caspase 3; CC7: cleaved caspase 7; CC8: cleaved caspase 8; CC9: cleaved caspase 9; 8-oxodG: 8-oxo-2′-deoxyguanosine; *γ*H2AX: H2A histone family member X; NQO1: NAD(P)H:quinone oxidoreductase 1; Bcl-2: B-cell lymphoma-2; Bax: Bcl-2-associated X protein; ATP: adenosine-triphosphate; CDK1: cyclin-dependent kinase 1; c-PARP: cleaved PARP; Bmi-1: B-lymphoma Mo-MLV insertion region 1; p21: protein 21; DNA-PKcs: DNA-dependent protein kinase, catalytic unit; NBS1: Nijmegen breakage syndrome 1; RAD51: radioresistance protein 51; TXNIP: Trx-interacting protein.

**Table 4:** Natural products modulating ROS in chemo-/radiotherapy of HNC.

Category	Herb	Site	Experimental model	Effective dose	Cotherapy	ROS detection	Biological effect	Mechanisms	Reference
Flavonoids	Quercetin	Larynx	In vitro (Hep2 cell)	40 *μ*M	+Cisplatin (2.5 *μ*g/ml)	—	Synergistic effects	↓Cu/Zn SOD, ↓p-AKT, ↑p-JNK, ↑c-FOS, ↓Bcl-2, ↓Bcl-xL, ↓survivin, ↑Bax, ↑cytochrome *c*, ↑caspase-3, ↑caspase-9, ↓NOS, ↓HSP70, ↓Ki-67, ↓telomerase	[216]
	Naringin	Esophagus	In vitro (YM1 cancer stem cell) In vivo (YM1 xenograft mouse)	In vitro (354 *μ*M) In vivo (50 mg/kg)	+Doxorubicin	—	Reduce side effect, restore the antioxidant defense system	↑SOD	[217]
	AIF	Esophagus	In vitro (Eca109, KYSE-30 cells) In vivo (Eca109 xenograft nude mice)	In vitro (5 *μ*M) In vivo (20 mg/kg/day)	+Radiation In vitro (6 Gy) In vivo (2 Gy/min on days 10 and 30)	DCFH-DA confocal microscope	Synergistic effects: enhance apoptosis G_2_/M arrest	↑ROS, ↓Nrf2, ↓HO-1, ↓NQO1, *γ*↑H2AX	[218]
	Wogonin	Larynx	In vitro (HN2, -HN3, -HN4, -HN5, and -HN9; SNU-1041, SNU-1066, and SNU-1076; HN4-*cis*R and HN9-*cis*R cells; normal cell: HOK-1, HOF, and HEK) In vivo (HN4-*cis*R or HN9-*cis*R xenograft nude mice)	50 mg/kg	+Cisplatin	DCFH-DA flow cytometry	Synergistic effects: enhance apoptosis	↑ROS, ↓GSH, ↓Nrf2, ↓GST, ↑p53, ↑p-JNK, ↑c-PARP, ↑PUMA	[219]

Polyphenols	Curcumin	Oral cavity	In vitro (normal cell SGNs and cancer cell PE/CA-PJ15) In vivo (male adult Wistar rats)	In vitro (3.37, 6.75 *μ*M) In vivo (200 mg/kg)	+Cisplatin	—	Otoprotective effect: antioxidant activity Synergistic effects: prooxidant and anti-inflammatory	Protective mechanisms: ↑Nrf2, ↑HO-1, ↓p53, ↓NF-*κ*B Synergistic mechanisms: ↓Nrf2 (nuclear), ↓NF-*κ*B, ↓pSTAT-3, ↑p53	[223]
	Curcumin	Pharynx	In vitro (HPV-cells: FaDu, SQ20B, JHU-022, HEK-001, and MSK-Leuk1; HPV+ cells: UPCI-SCC090 and UPCI-SCC154) In vivo (FaDu xenograft nude mice)	In vitro (10 *μ*M) In vivo (10 *μ*M)	+Radiation In vitro (0, 2, 4, 6 Gy) In vivo (0, 2, 4, 6 Gy)	—	Synergistic effects: inhibition of antioxidant defense system	↓TxnRd1	[145]
	FA	Oral cavity	In vitro (normal cell SGNs and cancer cell PE/CA-PJ15) In vivo (male adult Wistar rats)	In vitro (100-600 *μ*M) In vivo (600 mg/kg)	+Cisplatin	—	Otoprotective effect: antioxidant activity Synergistic effects: prooxidant at lower concentrations (100-600 *μ*M) Antagonistic effect: antioxidant at higher concentrations (>600 *μ*M)	Protective mechanisms: ↑Nrf2, ↑HO-1, ↓P53 Synergistic mechanisms: ↓Nrf2 (nuclear), ↓pSTAT-3 Antagonistic mechanisms: ↑Nrf2 (nuclear), ↑pSTAT-3	[223]
	DPP-23	Larynx	In vitro (HN3, HN3-*cis*R, HN4, HN4-*cis*R, HN9, HN9-*cis*R, HOK-1 cells) In vivo (HN9-*cis*R xenograft nude mice)	In vitro (2-40 *μ*M) In vivo (10 mg/kg)	+Cisplatin In vitro (10 *μ*M) In vivo (5 mg/kg)	DCFH-DA flow cytometry	Synergistic effects: inhibition of antioxidant defense system and activation of apoptosis	↑ROS, ↓GSH, ↓Nrf2, ↓HO-1, ↑p53, ↑c-PARP, ↑p21	[224]
	EGCG	Oral cavity	In vitro (normal cell: NHOK; cancer cell: HSC-2)	50-100 *μ*M	+Doxorubicin 0.625-5 *μ*M	DCFH-DA fluorescence microscope	Chemoprotective effect	↓ROS	[225]
	TA	Oral cavity	In vitro (normal cell: NHOK; cancer cell: HSC-2)	12.5-50 *μ*M	+Doxorubicin 0.625-5 *μ*M	DCFH-DA fluorescence microscope	Chemoprotective effect	↓ROS	[225]
	Epicatechin	Oral cavity	In vitro (oral fibroblast cells) In vivo (Zebrafish)	In vitro (50 *μ*M) In vivo (200 *μ*M)	+Radiation In vitro (20 Gy) In vivo (20 Gy)	DCFH-DA flow cytometry	Radioprotective effect: reduce apoptosis and restore MMP	↓ROS, ↓p38, ↓p-JNK, ↓CC3	[226]
	Epicatechin	Oral cavity	In vitro (human keratinocyte HaCaT cell) In vivo (Sprague-Dawley rats)	In vitro (100 *μ*M) In vivo (2 mM/day after radiation for 23 days)	+Radiation In vitro (20 Gy) In vivo (30 Gy)	DCFH-DA flow cytometry	Radioprotective effect	↓ROS, ↓p-JNK, ↓p38, ↓CC3, ↓NOX3	[227]

Quinones	Plumbagin	Tongue	In vitro (CAL27 cell, cisplatin-resistant cell line CAL27/CDDP) In vivo (CAL27/CDDP xenograft nude mice)	In vitro (5 *μ*M) In vivo (3 mg/kg every two days)	+Cisplatin In vitro (16.7 *μ*M) In vivo (4 mg/kg every three days)	DCFH-DA+MitoSOX fluorescence microscope	Synergistic effects: enhance apoptosis and autophagy	↑ROS, ↓Bcl-2, ↑Bax, ↑CC3, ↑Beclin-1, ↓p62, ↑LC-II/LC-I, ↓p-AKT, ↓p-mTOR, ↑p-JNK	[238]
	*β*-Lapachone	Head and neck	In vitro (FaDu, Detroit 562, SqCC/Y1, UMSCC-10A) In vivo (SqCC/Y1 xenograft SCID-NOD mice clinical samples)	In vitro (2.5 *μ*M) In vivo (10 mg/kg every other day)	+Radiation In vitro (2 Gy) In vivo (10 Gy)	DCFH-DA flow cytometry	Synergistic effects: enhance apoptosis and NDA damage	↓NQO1, ↑ROS, ↓Bcl-2, ↓ATP, ↑*γ*H2AX	[233]

Terpenoids	Oridonin	Larynx	In vitro (Hep-2 and Tu212 cells) In vivo (Hep-2 xenograft nude mice)	In vitro (12, 24, and 36 *μ*M) In vivo (20 mg/kg)	+Cetuximab In vitro (10 *μ*g/ml) In vivo (1 mg/mice)	DCFH-DA flow cytometry	Synergistic effects: enhance apoptosis and G_2_/M arrest	↑ROS, ↑CC8, ↑CC3, ↑c-PARP, ↑p21, ↑Fas, ↑FADD, ↑FasL, ↓ICAD, ↓cyclin B1, ↑p-cdc2, ↑p-cdc25c, ↓NAC, ↓CAT, ↑p-JNK, ↓p-EGFR	[244]

Ginsenosides	Ro	Esophagus	In vitro (ECA-109, TE-1 cell)	50 *μ*M	+5-Fluorouracil 100 *μ*g/ml	—	Synergistic effects: enhance DNA repair and inhibit autophagic flux	↑ESR2, ↑NCF1, ↑ATG-7, ↑CC3, ↑CC9, ↑c-PARP, ↑p62, ↓LC3BII/LC3BI, ↑CHEK1	[248]
	KRG	Oral cavity	In vitro (normal keratinocyte HaLa cell, cancer SCC25, SCC1484 cell) In vivo (zebrafish)	In vitro (10-100 *μ*g/ml) In vivo (30 *μ*g/ml)	+Radiation In vitro (8 Gy) In vivo (20 Gy)	DCFH-DA flow cytometry	Radioprotective effect	↓ROS, ↓ATM, ↓p-p53, ↓p-JNK, ↓p-p38, ↓CC3	[249]

*Note*. ROS: reactive oxygen species; DCFH-DA: 2′,7′-dichlorofluorescein diacetate; SOD: superoxide dismutase; AKT: protein kinase B; p-AKT: phosphorylated AKT; JNK: c-Jun N-terminal kinase; p-JNK: phosphorylated-JNK; c-FOS: cellular oncogene fos; Bcl-2: B-cell lymphoma-2; Bcl-xL: B-cell lymphoma-extra large; Bax: Bcl-2-associated X protein; NOS: nitric oxide synthase; HSP70: heat shock protein 70; AIF: alpinumisoflavone; Nrf2: nuclear factor (erythroid-derived 2)-like 2 transcription factor; HO-1: heme oxygenase; NQO1: NAD(P)H:quinone oxidoreductase 1; *γ*H2AX: H2A histone family member X; GSH: glutathione; GST: glutathione-S-transferases; p53: protein 53; c-PARP: cleaved poly-ADP ribose polymerase; PUMA: p53 upregulated modulator of apoptosis; NF-*κ*B: nuclear factor kappa-B; STAT: signal transducer and activator of transcription; TxnRd1: thioredoxin reductase 1; FA: ferulic acid; p21: protein 21; EGCG: epigallocatechin gallate; TA: tannic acid; CC3: cleaved caspase 3; NOX3: triphosphopyridine nucleotide oxidase 3; p62: sequestosome-1; LC3-II: light chain 3 II; LC3-I: light chain 3 I; p-mTOR: phosphorylated mammalian target of rapamycin; CC8: cleaved caspase 8; FADD: Fas-associated death domain; FasL: Fas ligand; ICAD: caspase-3-activated DNase inhibitor; p-cdc2: phosphorylated cdc2; p-cdc25c: phosphorylated cdc25c; NAC: N-acetyl cysteine; CAT: catalase; EGFR: epidermal growth factor receptor; p-EGFR: phosphorylated EGFR; Ro: ginsenoside Ro; ESR2: estrogen receptor 2; NCF1: neutrophil cytosolic factor 1; ATG-7: autophagy-related 7; CC9: cleaved caspase 9; CHEK1: checkpoint kinase 1; KRG: Korean red ginseng; p-p53: phosphorylated p53.

**Table 5:** Combination treatment of PDT with chemotherapy drugs in HNC.

Site	Model	Photosensitizer/laser irradiation	Cotherapy	ROS detection	Effect	Mechanisms	Reference
Larynx	In vitro (AMC-HN3 cell) In vivo (AMC-HN3 xenograft nude mice	Radachlorin (0.9 J/cm^2^)	+Carboplatin	DCFH-DA confocal microscope, flow cytometry	Synergistic effects: reduce side effect	↑cytochrome *c* ↓EGFR, ↑ROS	[258]

Larynx	In vitro (Hep-2 cell)	mTHPC (2 J/cm^2^)	+Cisplatin (5 *μ*M)	—	Synergistic effects	↓Bcl-2, ↓PD-L1 ↓ATG-7, ↓LC3-II/LC3-I	[259]

Oral cavity	In vitro (BHY cell)	mTHPC (1.8 J/cm^2^)	+Oxaliplatin (0.1-100 *μ*M)	DCFH-DA flow cytometry	Synergistic effects	↑ROS, ↑S-phase arrest	[260]

Esophagus	In vitro (KYSE-70 cell)	mTHPC (1.8 J/cm^2^)	+Cisplatin (0.01-50 *μ*M)	DCFH-DA flow cytometry	Synergistic effects	↑ROS	[260]

Head and neck	In vitro (cisplatin-resistant SQ20B and JSQ3 cell and cisplatin-sensitive HNSCC135 and SCC61 cell) In vivo (SQ20B xenograft nude mice)	Pyrolipid (54 J/cm^2^)	+Cisplatin (0.5 mg/kg)	—	Synergistic effects: enhance apoptosis	↑IL-6, ↑TNF-*α*, ↑IFN-*γ*	[261]

*Notes*. DCFH-DA: 2′,7′-dichlorofluorescein diacetate; mTHPC: *meta*-Tetra (hydroxyphenyl) chlorin; EGFR: epidermal growth factor receptor; Bcl-2: B-cell lymphoma-2; ROS: reactive oxygen species; LC3: microtubule-associated protein light chain 3; ATG-7: autophagy-related 7; TNF-*α*: tumor necrosis factor-*α*; IL-6: interleukin-6; IFN-*γ*: interferon-*γ*.
